# Lipidomic and Metabolomic Profiling on Low-Count Human Spermatozoa: A Robust and Reproducible Method for Untargeted HPLC-ESI-MS/MS-Based Approach

**DOI:** 10.3390/cells15070649

**Published:** 2026-04-05

**Authors:** Irune Calzado, Manu Araolaza, Mikel Albizuri, Ainize Odriozola, Iraia Muñoa-Hoyos, Iratxe Ajuria-Morentin, Nerea Subirán

**Affiliations:** 1 Faculty of Medicine and Nursing, University of the Basque Country, 48940 Leioa, Bizkaia, Spain; 2 Bizkaia Health Research Institute, 48903 Barakaldo, Bizkaia, Spain; 3 Galdakao-Usansolo Hospital, 48960 Galdakao, Bizkaia, Spain

**Keywords:** lipidomics, male infertility, metabolomics, MS/MS, sperm

## Abstract

**Highlights:**

**What are the main findings?**

- An optimized HPLC–ESI–MS/MS workflow enables comprehensive untargeted lipidomic and metabolomic profiling from only 1.25 million human sperm cells.
- The method achieved unprecedented coverage, identifying 473 lipids and 955 annotated metabolites, providing a detailed view of sperm membrane lipids, fatty acids, and metabolic pathways.

**What are the implications of the main findings?**

- This workflow enables high-coverage lipidomic and metabolomic analysis from minimal cell input, overcoming limitations related to sample availability.
- It provides a robust platform for future biomarker discovery and clinical applications in male infertility diagnostics.

**Abstract:**

Human infertility affects approximately 17.5% of the global population, with male factors accounting for nearly half of all cases. Identifying reliable molecular biomarkers is crucial for improving the diagnosis and assessment of male fertility. This study established and refined an untargeted high-performance liquid chromatography–electrospray ionization–tandem mass spectrometry (HPLC-ESI-MS/MS) protocol for a comprehensive lipidomic and metabolomic analysis of human spermatozoa, using only 1.25 million cells per sample. Compared with previous reports, our optimized method achieved an unparalleled level of analytical depth, identifying 473 lipid species and 955 structurally annotated metabolites. This corresponds to nearly a 7600-fold improvement in detection efficiency per cell compared with previously published approaches. Lipidomic analysis revealed that the most abundant lipid classes were glycerophospholipids (39%), cholesterol (20%) and fatty acids (19%), with cholesterol representing the single most abundant compound. This observation is consistent with the structural complexity of the sperm plasma membrane. Metabolomic profiling similarly identified glycerophospholipids (44%), eicosanoids (14%) and N-acyl amino acids (12%) as the major metabolite classes. The integration of lipidomic and metabolomic data highlighted functionally interconnected pathways related to membrane dynamics, energy metabolism, and hormone biosynthesis. Overall, this work establishes a robust, sensitive, and scalable analytical framework that enables the high-coverage molecular characterization of spermatozoa from limited sample material, laying the groundwork for future biomarker discovery and clinical applications in male infertility research.

## 1. Introduction

Human infertility is a significant global health and social issue, affecting approximately 17.5% of the world’s population, with male factors contributing to about half of all the cases [1]. This underscores the urgent need for reliable biomarkers to improve the assessment of male fertility. In this context, advanced omics technologies—including genomics, proteomics, and more recently, lipidomics and metabolomics—have enabled the identification of robust indicators of semen quality [2].

In terms of the technique, both nuclear magnetic resonance (NMR) and mass spectrometry (MS)-based methods have been developed for the study of lipidomics and metabolomics [3]. NMR spectroscopy offers several advantages, such as minimal sample preparation and non-destructive analysis of biological samples [4]. However, MS-based lipidomics and metabolomics have emerged as the most widely used techniques because of their high sensitivity, broader metabolite coverage and capacity to identify small variations in metabolite levels [5,6]. Among mass spectrometry (MS) platforms, liquid chromatography combined with mass spectrometry (LC-MS) is now the most widely used approach, although gas chromatography–mass spectrometry (GC-MS), direct infusion mass spectrometry (DI-MS), and flow injection analysis mass spectrometry (FIA-MS) have also been employed [7,8,9,10,11,12,13]. GC-MS is very effective for analyzing volatile and thermally stable metabolites. However, it frequently necessitates chemical derivatization to enhance volatility and detectability, hence constraining its applicability to specific compound classes [3]. Consequently, LC-MS has emerged as the favored method in metabolomics due to the absence of required sample derivatization, enabling the measurement of metabolites with different chemical structures and larger molecular sizes [3,7]. On the other hand, DI-MS and FIA-MS offer rapid, high-throughput analysis with minimal chromatographic separation, but are more susceptible to ion suppression and typically provide lower metabolite coverage and structural resolution than LC-MS-based approaches [14,15].

To date, most of the lipidomic and/or metabolomic studies in the context of male infertility have focused on seminal plasma, a seminal fraction that is essential for sperm viability and for supporting optimal fertilization conditions within the female reproductive tract [16]. Accordingly, seminal plasma has been extensively characterized using a variety of analytical platforms, predominantly HPLC-MS/MS [4,17,18,19,20,21,22], as well as GC-MS [8,9], NMR [23,24,25,26] and FIA-MS [10]. Conversely, fewer studies have explored the lipidomic and metabolomic profiles of spermatozoa, using HLPC-MS/MS [27,28], DI-MS [11], FIA-MS [12], NMR [6] and GC-MS [6,13]. This limited number of studies is largely due to the methodological challenges presented by spermatozoa when compared with seminal plasma. For example, in seminal plasma, the protein concentration varies between 35 and 55 g/L, while in spermatozoa, it is significantly lower, presenting additional challenges for both identification and quantitative analyses [24]. The reduced protein abundance in sperm samples often requires the use of a large number of cells to obtain sufficient analytical signal. This is a rather difficult task when the cell number is limited, and the quantification needs to be set to a higher threshold level.

Therefore, new approaches that enable high-quality extraction from a small number of spermatozoa are needed. In this study, we aimed to develop a reliable and reproducible untargeted HPLC-ESI-MS/MS-based workflow for the comprehensive lipidomic and metabolomic profiling of human spermatozoa. Our results represent an unprecedented increase in lipid and metabolite recovery relative to cell number, establishing a technical foundation for large-scale clinical applications, particularly in cases of limited sperm availability.

## 2. Materials and Methods

### 2.1. Sample Collection

The study was conducted in accordance with the principles outlined in the Declaration of Helsinki and was approved by the Ethics Committee of the Basque Country (CEImE: PI2019184). Semen samples were collected at Galdakao-Usansolo Hospital (Biscay) from the male partners of couples undergoing assisted reproductive treatments (IVF/ICSI) at the Assisted Reproduction Units of the Basque Public Health Service (Osakidetza, Vitoria-Gasteiz, Spain). All participants provided written informed consent prior to sample collection. Eligible participants were between 30 and 50 years of age, had a normal karyotype, no hormonal disorders, no physical abnormalities of the reproductive system, and no history of radiotherapy or chemotherapy. Only samples meeting the inclusion criteria and with signed informed consent were included in the study. Ejaculates were collected following a period of 3 to 5 days of sexual abstinence. A routine semen analysis was conducted according to the WHO standards [29] criterion to assess the sperm concentration (×10^6^ sperm/mL), using an improved Neubauer hemocytometer chamber. The sperm motility and kinematic parameters were assessed by using Computer-Assisted Sperm Analysis (CASA) 6.6.55 software and the sperm morphology was assessed using the SpermFunc^®^ Diff-Quick Staining Kit (CellaVision, Lund, Sweden). Subsequently, multiple washing steps were conducted to separate seminal plasma from spermatozoa and to eliminate other cell types. The samples were then stored at −80 °C until further molecular analysis.

### 2.2. Sample Preparation and Extraction for Lipidomics

Spermatozoa from three pooled biological samples (5 M cells) were used for method development. The pooled sample was generated by combining equal numbers of spermatozoa obtained from three independent ejaculates. Spermatozoa were then sonicated in two 2.5 million cell aliquots on a Vibra Cell sonicator (Bioblock Scientific, Illkirch, France) equipped with a 3 mm A-23 probe at 75% amplitude for 7 cycles of 20 s ON/20 s OFF. Following sonication, each aliquot was further divided into two equal parts (1.25 M cells), generating four technical replicates for downstream lipidomics analysis (Supplementary Figure S1). The protein concentration of each aliquot was measured using the BCA^TM^ Protein Assay Kit (Thermo Scientific, Rockford, IL, USA).

For the lipid extraction, 10 µL of a selected class-specific internal standard mixture (Splash™ LipidoMix™, Cer/Sph Mixture I, Cardiolipin Mix I and 24:0 (d4) L-carnitine, Avanti Polar Lipids, Inc., Alabaster, AL, USA; Supplementary Table S1) was added, followed by four volumes of pre-cooled isopropanol (IPA) at −20 °C. The samples were then vortexed on a Vortex V-1 plus (Biosan, Riga, Latvia) for 1 min, incubated at room temperature for 10 min, and centrifuged at 13,000× *g* for 20 min at 4 °C. The resulting supernatants were dried using a SpeedVac Concentrator SC250EXP (Thermo Savant, Holbrook, NY, USA) for approximately 2 h and stored in an inert atmosphere at −80 °C.

### 2.3. Sample Preparation and Extraction for Metabolomics

In parallel with lipidomics, spermatozoa from three biological samples (5 M cells) were used for method development. The pooled sample was generated by combining equal numbers of spermatozoa obtained from three independent ejaculates and then pooled spermatozoa was sonicated in two 2.5M-cell aliquots on a Vibra Cell sonicator (Bioblock Scientific), as described above (Supplementary Figure S1). Similarly to lipidomics, four technical replicates (1.25 M cells) were generated, and the protein concentration of each sample was measured using the BCA Protein Assay Kit (Thermo Scientific). For metabolomics, metabolites extractions were carried out using three volumes of pre-cooled methanol (−20 °C). The samples were then vortexed for one minute, incubated at room temperature for ten minutes, and then centrifuged at 13,000× *g* for twenty minutes at 4 °C. The samples were then dried and stored under an inert atmosphere at −80 °C.

### 2.4. Lipidomics Analysis by HPLC-ESI-MS/MS

The lipid extracts were analyzed using a reverse-phase UHPLC-QOrbitrap-HFX system equipped with an Acquity™ Premier C18 CSH VanGuard™ column (2.1 × 100 mm, 1.7 µm, Waters Corporation, Milford, MA, USA). The columns were pre-equilibrated and cleaned with a water/methanol/formic acid solution (95:5:0.1, *v*/*v*) at a flow rate of 100 µL/min. The samples were resuspended in 70 µL of acetonitrile:isopropanol (50:50, *v*/*v*) solution, vortexed for 2 min and 30 s, ultrasonicated for 1 min, and centrifuged at 13,000 rpm for 4 min at 4 °C. Analyses were conducted in both positive and negative electrospray ionization modes (ESI), after injecting 2 µL for ESI+ mode and 5 µL for ESI− mode.

### 2.5. Metabolomics Analysis by HPLC-ESI-MS/MS

The metabolic extracts were analyzed using a reverse-phase UHPLC-QOrbitrap-HFX system equipped with an Acquity™ UPLC BEH C18 WATERS™ column (2.1 × 100 mm, 1.7 µm, Waters Corporation, Milford, MA, USA). The columns were pre-equilibrated and cleaned as described in the section above. Samples were resuspended in 70 µL of water:acetonitrile (10:90, *v*/*v*) solution and then vortexed for 2 min and 30 s, ultrasonicated for 1 min, and centrifuged at 13,000 rpm for 4 min at 4 °C. Analyses were conducted in both positive and negative electrospray ionization modes (ESI), but in this case, the injection volume was 2 µL for both ionization modes.

### 2.6. Data Processing and Statistical Analysis

The raw data obtained for the lipidomic analysis was processed with Lipid Search^TM^ 4.2.27 software (Thermo Fisher Scientific, Waltham, MA, USA). The key processing parameters applied were: target database, General; precursor tolerance, 5 ppm; product tolerance, 5 ppm; product ion threshold, 1%; m-score threshold, 2; Quan *m*/*z* tolerance, ±5 ppm; Quan RT (retention time) range, ±0.5 min; use of main isomer filters and ID quality filters A, B, C and D; and Adduct ions H^+^, Na^+^ and NH_4_^+^ for positive ion mode, and H^−^ and HCOO^−^ for negative ion mode. The lipid classes determined were: Acylcarnitine (AcCa), ceramide (Cer), cholesterol (Chol), diacylglycerol (DG), fatty acids (FA), monohexosylceramide (Hex1Cer), dihexosylceramide (Hex2Cer), trihexosylceramide (Hex3Cer), lysophosphatidylcholine (LPC), lysophosphatidylcholine (ether/plasmalogen) (LPCe), lysophosphatidylethanolamine (LPE), lysophosphatidylglycerol (LPG), lysophosphatidylserine (LPS), phosphatidylcholine (PC), ether-linked phosphatidylcholine (PCe), phosphatidylethanolamine (PE), phosphatidylethanolamine (ether/plasmalogen) (PE(e/p)), phosphatidylglycerol (PG), phosphatidylinositol (PI), phosphatidylserine (PS), sphingomyelin (SM), sphingosine (SPH), sulfatide (ST) and triacylglycerol (TG).

After processing, the lipidomics data matrix was refined by evaluating correlations between the carbon number and the number of double bonds within each lipid class. The linearity was assessed by calculating the correlation coefficient (R^2^) across different injection volumes, with acceptable values defined as R^2^ > 0.800. The quantification was conducted by normalization of the intensity of the monoisotopic peak of each native lipid species, with respect to the intensity of the corresponding monoisotopic peak of the internal standard from Splash LipidoMix, Cer/Sph Mixture I, Cardiolipin Mix I and 24:0 (d4) L-carnitine (Avanti Polar Lipids, Inc.) (Supplementary Table S1). The final values were then normalized to the protein concentration and expressed in nmol/mg of protein. Lastly, enrichment analysis was carried out using the MetaboAnalyst 6.0 software and the SMPDB (The Small Molecule Pathway Database).

For metabolomics, Compound Discoverer 3.2 (Thermo Fisher Scientific, Waltham, MA, USA) was used to process the raw data, following the “untargeted metabolomics with statistics detect unknowns and ID using online databases” workflow. All identifications were based on accurate mass and MS/MS spectral matching against online databases (Predicted Compositions, mzCloud Search, Metabolica Search, ChemSpider Search and MassList Search). After processing, the initial data matrix was filtered based on the database match results, applying the following criteria: “Full Match”, “No Results”, “Not the Top Hit” and “Partial Match.” Annotations were accepted when at least one database provided a “Full Match”, indicating high spectral similarity and compositional agreement. From the resulting data matrix, further cleaning was performed by calculating the correlation coefficient (R^2^) across different injection volumes, with acceptable values defined at R^2^ > 0.800. Finally, the (*m*/*z*) values were normalized to mg of protein (expressed as (*m*/*z*)/mg protein). Similarly, enrichment analysis was carried out in the software MetaboAnalyst 6.0, using the SMPDB (The Small Molecule Pathway Database).

## 3. Results

### 3.1. Optimization of Sonication Process

Three biological samples obtained from patients undergoing assisted reproductive treatment at Galdakao-Usansolo Hospital were used for lipidomic and metabolomic analyses. The patients were aged between 33 and 35 years, and had abstained from sexual activity for 4–5 days prior to sample collection. According to the reference values described by WHO (2021), the samples were classified as normozoospermia (C: 195 M/mL; TSC: 362 M; PR: 60%; TMS: 70%; M: 31% and V: 86%), teratozoospermia (C: 200 M/mL; TSC: 400 M; PR: 31%; TMS: 46%; M: 0.91% and V: 66%) and asthenoteratozoospermia (C: 26 M/mL; TSC: 85 M; PR: 10%; TMS: 18%; M: 12% and V: 71%) (Supplementary Table S2). The kinematic parameters were also evaluated. The normozoospermic sample exhibited higher velocity values (VCL, VSL, VAP), linearity indices (LIN, STR, WOB) and beat cross frequency (BCF) compared to pathological samples. No hyperactivated spermatozoa were observed in any of the three samples (Supplementary Table S2), which is characteristic for non-capacitated spermatozoa.

Prior to the lipidomic and metabolomic analyses, the ultrasonication parameters used in this study were optimized in a stepwise manner by evaluating the effects of sonication intensity, number of cycles, and cycle duration on sperm cell disruption efficiency. First, based on the protocols previously described in the literature [30,31], sonication intensities ranging from 25% to 75% were tested using three cycles of 10 s ON/10 s OFF, with aliquots containing approximately 1 million sperm cells (Supplementary Table S3). All experiments were performed on ice to prevent sample overheating and the degradation of temperature-sensitive molecules. Cell disruption efficiency was assessed by microscopic evaluation, examining the proportion of intact spermatozoa compared with separated heads and tails (Supplementary Figure S2). Under these conditions, effective sperm disruption was consistently observed at only 75% intensity, whereas lower intensities resulted in the persistence of intact sperm cells (Supplementary Table S3). In the second step, the intensity was maintained at 75%, and the number of cycles (three, five, and seven cycles) was evaluated. All tested conditions resulted in detectable sperm disruption (Supplementary Table S4). However, to further assess the extraction efficiency, additional experiments were performed using aliquots containing 2.5 million sperm cells (Supplementary Table S5). In these experiments, different cycle durations (10 s and 20 s ON/OFF) and cycle numbers were tested, and the cell disruption was evaluated by both microscopic observation and protein concentration measurement (BCA assay). The results indicated that longer cycles (20 s ON/OFF) and a greater total sonication time increased the likelihood of complete sperm cell disruption. In contrast, shorter cycles or lower total sonication times resulted in inconsistent disruption. Conditions using ≥5 cycles with 20 s ON/OFF intervals showed improved efficiency, with the most effective results obtained at 75% amplitude with seven cycles of 20 s ON/20 s OFF performed on ice.

### 3.2. Lipidomic Profiling of Human Spermatozoa

**Figure 1:**
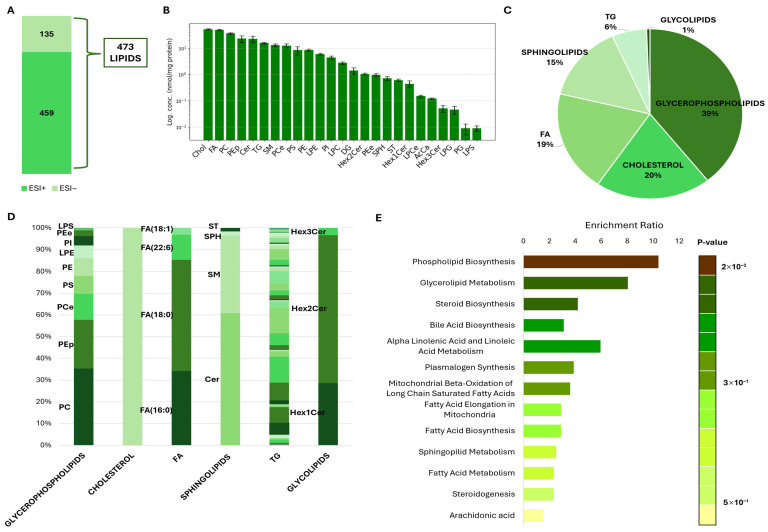
Lipidomics overview in human spermatozoa. (**A**) Feature detection in spermatozoa by untargeted lipidomics. The bar plots show the number of lipids identified in positive (ESI+) and negative (ESI−) ionization modes. The datasets were merged, and the unified dataset with 473 lipids was retained for further analyses. (**B**) Lipid class concentrations in spermatozoa, normalized to protein concentration (nmol/mg protein) and expressed in logarithmic scale (Acylcarnitine (AcCa), ceramide (Cer), cholesterol (Chol), diacylglycerol (DG), fatty acids (FA), monohexosylceramide (Hex1Cer), dihexosylceramide (Hex2Cer), trihexosylceramide (Hex3Cer), lysophosphatidylcholine (LPC), lysophosphatidylcholine (ether/plasmalogen) (LPCe), lysophosphatidylethanolamine (LPE), lysophosphatidylglycerol (LPG), lysophosphatidylserine (LPS), phosphatidylcholine (PC), ether-linked phosphatidylcholine (PCe), phosphatidylethanolamine (PE), phosphatidylethanolamine (ether/plasmalogen) (PE(e/p)), phosphatidylglycerol (PG), phosphatidylinositol (PI), phosphatidylserine (PS), sphingomyelin (SM), sphingosine (SPH), sulfatide (ST) and triacylglycerol (TG)). (**C**,**D**) Distribution of major lipid categories identified in spermatozoa, grouped into: glycerophospholipids (LPC, LPCe, LPE, LPG, LPS, PC, PCe, PE, PEe, Pep, PG, PI, PS; 39%), sterols (cholesterol; 20%), fatty acids (FA; 19%), sphingolipids (Cer, SM, SPH, ST; 15%), triacylglycerols (TG; 6%) and glycolipids (Hex1cer, Hex2Cer, Hex3cer; 1%). (**E**) Lipid set enrichment analysis of the lipidomic dataset performed in MetaboAnalyst, using the Small Molecule Pathway Database (SMPDB). The bar plot displays the top enriched metabolite sets ranked by enrichment ratio.

Untargeted lipidomic analysis revealed the presence of a broad range of lipid species present in human spermatozoa, identified in both positive (ESI+) (Supplementary Table S6A) and negative (ESI−) (Supplementary Table S6B) ionization modes. Precisely, we identified 459 lipids in ESI+ and 135 in ESI−, and after merging the two datasets, a unified lipid profile of 473 compounds was obtained (Figure 1A; Supplementary Table S6C).

To evaluate the efficiency of our workflow, we compared our dataset with the previous analytical platforms used for lipidomics profiling of human spermatozoa. Only four studies reporting broad lipid coverage in sperm cells are published to date (Table 1), showing high variability in both the number of identified lipids (ranging from 140 to 479 species) and the sperm cell counts used for extraction (from 5.46 to over 464 million). Using a combination of normal-phase (NP)-HPLC-MS and reverse-phase (RP)-HPLC-MRM-MS, a total of 479 lipids were identified from 10 million sperm cells, although the authors do not specify how many were detected with each technique. Using direct-infusion DI-ESI-MS for an average of 5.46 million sperm cells, the authors reported 399 species; and using a targeted FIA-MS/MS approach, the authors reported 140 lipid species from an average of 464 million spermatozoa. Our HPLC-ESI-MS/MS analysis identified 473 lipid species using only 1.25 million sperm cells, indicating the highest lipid coverage reported to date with the lowest cell number. In fact, when we normalized to the cell number, our workflow yielded an efficiency of 378.4 lipids per million sperm cells, which was markedly higher than the efficiencies reported in previous studies. The combined NP- and RP-HPLC-MS strategy achieved 47.9 lipids per million cells, whereas DI-ESI-MS reached 73.1 lipids per million and HILIC-HPLC-ESI-MS/MS 10.5 lipids per million, indicating that our method is approximately 8-fold, 5-fold, and 36-fold more efficient, respectively. The targeted FIA-MS/MS approach showed the lowest efficiency, with only 0.3 lipids per million spermatozoa. This underscores that our untargeted HPLC-ESI-MS/MS analysis provides a lipid identification density per cell that is more than three orders of magnitude greater.

Next, to evaluate the robustness of the method and its applicability to samples with limited sperm availability, we examined the lipid coverage across decreasing sperm concentrations, using the same pooled sample. In this context, lipidomic analysis was performed on two serial dilutions of cell concentration, 0.3125 M and 0.625 M, each analyzed in four replicates. Across the tested concentration range, the total number of confidently annotated lipids decreased with lower sperm concentrations, with 415, 443 and 473 species detected at 0.3125 M, 0.625 M and 1.25 M, respectively (Supplementary Figure S3A). Violin plots of individual lipid concentrations showed similar distributions at 0.625 M and 1.25 M sperm cells, and a broader asymmetric distribution at 0.3125 M (Supplementary Figure S3B). Venn diagrams of the four replicates per condition revealed a significant central overlap, indicating that most lipid species were consistently detected in all replicates at each sperm concentration (Supplementary Figure S3C). The lipid overlaps of the four replicates were 82.2%, 84.4% and 85.8% for 0.3125 M, 0.625 M and 1.25 M sperm cells, respectively. Although all three sperm concentrations yielded a higher number of lipid identifications than in previously reported studies, the 1.25 M concentration provided the highest lipid coverage and reproducibility. While the reproducibility across replicates did not reach 100%, due to analytical detection limits, this concentration was considered optimal for subsequent analyses. The observed overlap of 85.8% indicates that the majority of the sperm lipidome is consistently captured and suggests that this workflow can be reliably extended to samples with limited sperm availability.

After confirming the robustness and efficiency of the analytical workflow, we proceeded to characterize the qualitative composition of the human sperm lipidome. In our dataset, the detected lipids were classified into 25 major classes, as shown in Figure 1B, presented on a logarithmic scale and normalized to the protein concentration (nmol/mg protein). Overall, cholesterol (Chol) was the most abundant lipid species in spermatozoa, followed by fatty acids (FA), phosphatidylcholines (PC), phosphatidylethanolamine plasmalogens (PEp), and ceramides (Cer). Intermediate levels were observed for sphingomyelins (SM), triacylglycerols (TG), ether-linked phosphatidylcholines (PCe), phosphatidylethanolamines (PE), and phosphatidylserines (PS). In contrast, lysolipids (LPG and LPS), glycolipids (Hex1Cer and Hex3Cer), acylcarnitines (AcCa), and phosphatidylglycerol (PG) were detected at lower concentrations. These lipid classes together constitute the main classes normally described in plasma membrane lipids [32]: glycerophospholipids (LPC, LPCe, LPE, LPG, LPS, PC, PCe, PE, PEe, Pep, PG, PI, PS; 39%), sterols (cholesterol, 20%), sphingolipids (Cer, SM, SPH, ST, 15%), triacylglycerols (TG; 6%) and glycolipids (Hex1cer, Hex2Cer, Hex3cer, 1%) (Figure 1C).

Following this classification, Figure 1D shows the relative distribution within each lipid class. In the glycerophospholipid fraction, PC and PEp represent the most abundant subclasses, followed by PE, PS, PI, LPE, PCe, and LPS. The fatty acid (FA) pool is dominated by saturated palmitic acid (FA(16:0)) and stearic acid (FA(18:0)), with additional contributions from monounsaturated oleic acid (FA(18:1)) and the highly unsaturated docosahexaenoic (DHA) acid (FA(22:6)). Among sphingolipids, SM accounts for the largest proportion, with smaller fractions of Cer, SPH, and ST. The TG group shows a similar distribution of the compounds within this group. Glycolipids are mainly represented by Hex1Cer and Hex2Cer, with a smaller amount of Hex3Cer present.

Enrichment analysis performed on MetaboAnalyst using the Small Molecule Pathway Database (SMPDB) showed phospholipid biosynthesis, glycerolipid metabolism, and steroid biosynthesis as the most enriched pathways (Figure 1E). Additional over-represented pathways include bile acid biosynthesis, alpha-linolenic acid and linoleic acid metabolism, plasmalogen synthesis, mitochondrial beta-oxidation of long-chain saturated fatty acids, and fatty acid elongation and biosynthesis. Lower enrichment ratios are observed for sphingolipid metabolism, fatty acid metabolism, steroidogenesis, and arachidonic acid metabolism.

### 3.3. Metabolomic Profiling of Human Spermatozoa

**Figure 2:**
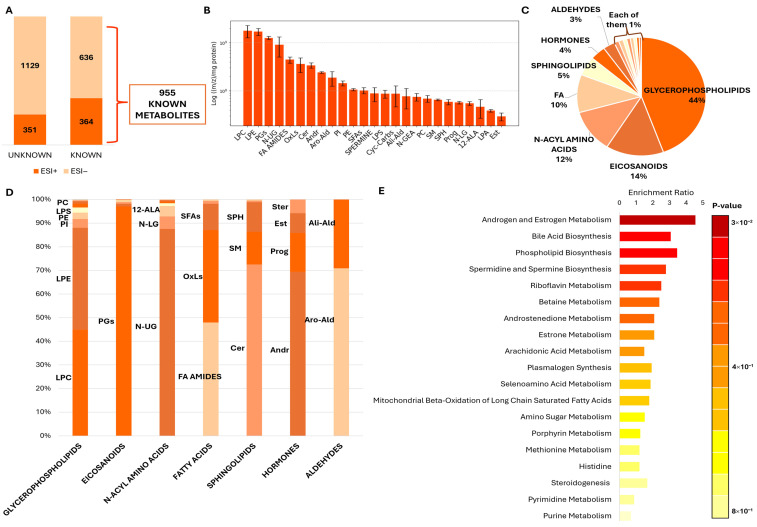
Metabolomics overview in human spermatozoa. (**A**) Feature detection in spermatozoa by untargeted metabolomics. The bar plots show the number of metabolites identified in positive (ESI+) and negative (ESI−) ionization modes, classified as known and unknown. The datasets were merged, and only the 955 known metabolites were retained for downstream analyses. (**B**) The top 25 metabolites and classes detected in spermatozoa. Relative concentrations are normalized to the protein concentration ((*m*/*z*)/mg protein) and expressed in logarithmic scale (12-aminolauric acid (12-ALA), androgens (Andr), aliphatic aldehydes (Ali-Ald), aromatic aldehydes (Aro-Ald), ceramides (Cer), cyclic carbohydrates (Cyc-Carbs), estrogens (Est), fatty acid amines (FA amides), lysophosphatidic acid (LPA), lysophosphatidylcholine (LPC), lysophosphatidylethanolamine (LPE), lysophosphatidylserine (LPS), N-gondoylethanolamine (N-GEA), N-lauroylglycine (N-LG), N-Undecanoylglycine (N-UG), oxylipins (OxLs), phosphatidylcholine (PC), phosphatidylethanolamine (PE), phosphatidylinositol (PI), progestogens (Prog), prostaglandins (PGs), saturated fatty acids (SFAs), sphingomyelin (SM), sphingosine (SPH) and spermine). (**C**,**D**) Classification and distribution of major metabolite categories identified in spermatozoa, grouped mainly into: glycerophospholipids (glycosylphosphatidylinositol (GPI), glycosylphosphatidylglycerol (Glyco-PG), LPA, LPC, LPE, PI, LPS, PC, PE, phosphatidylglycerol (PG), phosphatidylserine (PS); 45%), eicosanoids (docosahexaenoic acid (DHA) derivates, epoxydocosapentaenoic acid derivates (EDE), hydroxyeicosatetraenoic acid derivates (HETE), lypoxines (LX), PGs, thromboxanes (TX); 15%), N-acyl amino acids ((12:0) N-biotinyl, 12-ALA, N-[(4Z,7Z,10Z,13Z,16Z,19Z)-docosahexaenoyl]-L-glutamic acid, N-arachidonyl-L-alanine, N-arachidonoyl-gamma-aminobutyric acid (N-arachidonoylGABA), N-LG, N-Palmitoyl-L-tyrosine, N-UG; 12%), fatty acids (FA amides, monounsaturated fatty acids, OxLs, poly-unsaturated fatty acids (PUFAs), SFAs; 10%), sphingolipids (Cer, glycosphingolipid, lysosphingomyelin (Lyso SM), SM, SPH; 5%), hormonal metabolites (Andr, Est, Prog, steroids (Ster); 4%), aldehydes (Ali-Ald, Aro-Ald; 3%), peptides (1%), amines (1%), carbohydrates (1%), acylcarnitines (1%), amino alcohols (1%), acylcarnitines (1%), bile acids (1%), endocannabinoids (1%) and nucleosides (1%). (**E**) Metabolite-set enrichment analysis performed in MetaboAnalyst using the Small Molecule Pathway Database (SMPDB). The bar plot represents the top enriched metabolite sets, ranked by enrichment ratio.

In the untargeted metabolomic approach, a total of 715 features were detected in positive mode (ESI+), of which 364 were successfully annotated based on accurate MS/MS spectra and comparison with the database sets (Predicted Compositions, mzCloud Search, Metabolica Search, ChemSpider Search and MassList Search) (Supplementary Table S7A), while 351 remained unidentified, as no matches were found. Similarly, the negative mode (ESI−) yielded 1765 features, with 636 compounds annotated (Supplementary Table S7B) and 1129 remaining as unknowns. To ensure data robustness and biological interpretability, both ionization datasets were merged and curated, retaining only those metabolites that were confidently identified (with assigned names and valid database matches). A total of 955 compounds were successfully identified (Figure 2A; Supplementary Table S7C), and this unified dataset was then used for all subsequent analyses.

Following lipidomic analysis, to also evaluate the efficiency of our metabolomic workflow, we compared our dataset with previous published analytical platforms used for metabolomics profiling of human spermatozoa. Published metabolomic studies in human spermatozoa (Table 2) have reported far lower numbers of identified metabolites (ranging from 27 to 112 species), despite using a markedly higher cell concentration (ranging from 15 million to over 464 million). Specifically, HILIC-HPLC-ESI-MS/MS identified 112 metabolites from 20 million spermatozoa; GC-MS detected between 27 and 33 metabolites from an average of 15 and 102.8 million cells, respectively; NMR detected 42 metabolites from 150 million cells; and a targeted FIA-MS/MS workflow reported 31 metabolites from a median of 464 million sperm cells. Our HPLC-ESI-MS/MS analysis identified 2435 metabolic features, including 955 structurally annotated metabolites, using 1.25 million sperm cells. This represents the highest metabolomic coverage reported to date. Furthermore, when considering only structurally annotated compounds, our study achieved an efficiency of 764 metabolites per million sperm cells, indicating a markedly higher metabolite yield per cell than in previously reported approaches. In comparison, the HILIC-HPLC-ESI-MS/MS study reported 5.6 metabolites per million spermatozoa, the GC-MS analysis reported 1.8 and 0.3 metabolites per million cells, the NMR analysis reported 0.3 metabolites per million and the targeted FIA-MS/MS method reported only 0.1 metabolites per million. This shows that our method provides approximately 140-fold, 430-fold, 2500-fold, and 7600-fold higher annotation density per cell, respectively.

We also investigated the impact of the sperm concentration on the metabolomic coverage by analyzing the same pooled sample at reduced cell concentrations (0.3125 M and 0.625 M). In contrast to lipidomics, the metabolomic coverage was not affected by the sperm concentration, with the total number of confidently annotated metabolites remaining constant across all conditions, with 2435 metabolites detected in each case (Supplementary Figure S4A). However, the violin plots of individual metabolic concentrations showed similar profiles at 0.625 M and 1.25 M sperm cells, whereas a wider and more asymmetric distribution was evident at the lowest concentration (Supplementary Figure S4B), which was mainly due to the limited dynamic response of the analytical platform. The reproducibility across replicates was high for all conditions. Venn diagram analysis demonstrated a complete overlap of metabolite identifications among the four replicates at each sperm concentration (Supplementary Figure S4C). Overall, although all three sperm concentrations yielded the same number of metabolites with good reproducibility, the variability was lower at 1.25 M sperm cells. Therefore, this concentration was selected for subsequent analyses.

In this context, to characterize the metabolic composition of spermatozoa and given the large number of detected compounds, the metabolites were classified into classes. The 25 most abundant classes identified in our dataset are represented in Figure 2B, expressed on a logarithmic scale and normalized to the protein concentration ((*m*/*z*)/mg protein). Overall, lysophospholipids LPC and LPE were the most abundant metabolites in spermatozoa, followed closely by prostaglandins (PGs) and N-undecanoglycine (N-UG), an N-acyl amino acid derivative. Intermediate concentrations were observed for fatty acid (FA) amides, oxylipins (OxLs), ceramides (Cer), androgens (Andr) and aromatic aldehydes (Aro-Ald). In contrast, sphingolipids (SM and SPH), lysophosphatidic acid (LPA), the steroid classes progestogens (Prog) and estrogens (Est), and the N-acyl amino acids N-lauroylglycine (N-LG) and 12-aminolauric acid (12-ALA) were detected at lower concentrations.

The chemical classification of the known metabolites showed that spermatozoa are enriched in lipid-derived molecules, collectively representing more than three-quarters of the detected metabolome (Figure 2C,D). Glycerophospholipids represented the largest group (44%), including multiple subclasses, such as glycosylphosphatidylinositol (GPI), glycosylphosphatidylglycerol (Glyco-PG), LPA, LPC, LPE, PI, LPS, PC, PE, PG, and PS (Figure 2D). Eicosanoids constituted the second major category (14%), comprising DHA derivatives, epoxydocosapentaenoic acids (EDE), hydroxyeicosatetraenoic acids (HETE), lipoxins (LX), thromboxanes (TX), and especially prostaglandins (PGs). The latter were the most abundant compounds within this family. N-acyl amino acids formed 12% of the metabolome and included several long-chain conjugates, such as N-LG, and N-UG. Fatty acids, including saturated, monounsaturated, polyunsaturated, FA amides, and oxylipins, accounted for 10% of the dataset. Additional lipid-related groups included sphingolipids (Cer, GSL, Lyso SM, SM, SPH; 5%) and hormonal metabolites (androgens, estrogens, progestogens, steroids; 4%). Minor metabolite classes were also detected, each contributing 1–3% to the annotated metabolome. These included aldehydes (3%), peptides (1%), amines (1%), carbohydrates (1%), amino alcohols (1%), acylcarnitines (1%), bile acids (1%), endocannabinoids (1%) and nucleosides (1%).

Subsequent enrichment analysis performed on MetaboAnalyst revealed androgen and estrogen metabolism, bile acid biosynthesis, phospholipid biosynthesis and spermidine and spermine biosynthesis as the most enriched pathways (Figure 2E). Additional enriched pathways include riboflavin metabolism, betaine metabolism, androstenedione metabolism, estrone metabolism, arachidonic acid metabolism, plasmalogen synthesis, selenoamino acid metabolism and mitochondrial beta-oxidation of long chain saturated fatty acids. On the other hand, lower enrichment ratios were observed for amino sugar metabolism, porphyrin metabolism, methionine metabolism, histidine, steroidogenesis, pyrimidine metabolism and purine metabolism.

## 4. Discussion

This study presents the optimization of a robust, efficient and reproducible HPLC-ESI-MS/MS-based workflow for an untargeted lipidomic and metabolomic analysis of human spermatozoa. To obtain the most representative lipidomic and metabolomic profiles, we analyzed a combination of normozoospermic and pathological samples. An untargeted lipidomic and metabolomic approach was applied, using only 1.25 million sperm cells per sample, leading to the identification of 473 lipids and 955 metabolites, respectively. This represents the most comprehensive profiling achieved from such a limited number of sperm cells. Although 955 metabolites could be confidently annotated with names in our study, a total of 2,435 features have been detected alongside their respective retention times and *m*/*z* values. This is by far the highest number of metabolite identifications reported in sperm metabolomics to date, surpassing previous studies and highlighting the unprecedented coverage achieved with our workflow.

A critical methodological aspect lies in cell disruption, which can be a key factor contributing to the increase in our efficiency. In our procedure, we performed sonication prior to analysis, as sonication employs ultrasonic waves to disrupt cell membranes and release intracellular content, thereby outperforming homogenization in sperm extraction efficiency [31]. While sonication is becoming increasingly adopted, homogenization remains the procedure of choice in many laboratories [11,27], despite evidence suggesting that sonication produces enhanced biomolecule extraction in spermatozoa and provides greater extraction efficiency than homogenization [33]. Our data corroborates this, demonstrating that optimized sonication significantly improves both lipid and metabolite coverage. To address variability across the existing sperm lipidomic and metabolomic studies, we applied a double-normalization strategy, standardizing cell numbers and normalizing metabolite abundance to protein content. This reduced sample-to-sample variability and improved quantitative reliability. In most studies conducted on spermatozoa, cell numbers are not standardized [11,12,13], and proper normalization is frequently overlooked, making biological interpretation and comparison across studies difficult.

Regarding lipidomics, our results show that membrane lipids are the predominant lipid species in human spermatozoa, with cholesterol serving as a key structural and regulatory component influencing membrane rigidity, stability, and fluidity [34]. Enrichment analysis revealed cholesterol’s involvement in steroidogenesis, bile acid, and steroid hormone biosynthesis [35], while glycerophospholipids were linked to membrane assembly and remodeling [36]. The enrichment of phospholipid and glycerolipid metabolic pathways reflects the complex and dynamic nature of the sperm plasma membrane, which is characteristic for non-capacitated spermatozoa [37].

Fatty acids (FA) were among the most abundant lipid species detected. These molecules are key energy substrates and structural components of complex lipids [38,39,40], as evidenced by the enrichment analysis participating in pathways such as plasmalogen synthesis, mitochondrial β-oxidation of long-chain saturated fatty acids, fatty acid elongation, fatty acid biosynthesis and fatty acid metabolism. Furthermore, spermatozoa exhibited a predominance of long-chain fatty acids, including polyunsaturated species such as docosahexaenoic acid (DHA, 22:6) and oleic acid (18:1). Long-chain PUFAs incorporated into membrane phospholipids are synthesized from the essential dietary precursors linoleic and α-linolenic acids through elongation and desaturation reactions [41]. These long-chain derivatives contribute to maintaining optimal membrane fluidity and flexibility, which are critical during processes such as capacitation, acrosome reaction and sperm motility [37]. Moreover, PUFAs serve as precursors for bioactive lipid mediators, including prostaglandins and leukotrienes, which are derivatives of arachidonic acid, also shown in the enrichment analysis [41]. Triacylglycerols also showed relevance in the enrichment analysis, as they are involved in glycerolipid metabolism and act as energy substrates for β-oxidation through hydrolysis to release fatty acids [27].

Lastly, sphingolipid metabolism, which is involved in membrane signaling and cellular regulation [42,43], is likewise significantly enriched, supporting the functional relevance of the sphingolipids in human spermatozoa.

Metabolomic analysis confirmed the strong representation of lipid-associated biochemical pathways. Phospholipid biosynthesis emerged as one of the most significantly enriched pathways, in line with the high proportion of glycerophospholipids detected. On the other hand, the pathway with the highest enrichment ratio was androgen and estrogen metabolism, which can be integrated with other detected steroid-related pathways, including steroidogenesis, androstenedione and estrone metabolism. This further supports the presence of metabolites involved in the synthesis and regulation of sex hormones, which are essential for sperm maturation and function [44,45].

Pathways related to fatty acid metabolism (plasmalogen synthesis and mitochondrial β-oxidation of long-chain fatty acids) and their derivatives, such as arachidonic acid metabolism, were also enriched in our results. This reinforces the biological relevance of prostaglandins and other eicosanoids observed in the profiling [41,46], as well as the importance of fatty acids as energy substrates in non-capacitated spermatozoa.

Notably, amino acid- and amine-related processes (spermidine/spermine biosynthesis, betaine, selenoamino acid, methionine, histidine and purine metabolism) were also significantly enriched. Interestingly, bile acid biosynthesis was also significantly enriched, despite low absolute abundance. Bile acids are not synthesized directly by spermatozoa but rather originate mainly from accessory gland secretions, such as prostate or seminal vesicles [47,48]. Likewise, pyrimidine metabolism was determined with a low abundance, reflecting the presence of nucleosides. Finally, some enriched pathways were not captured by the chemical classification due to their low abundance, yet they still showed significant pathway enrichment; these include riboflavin metabolism (vitamin B2), amino sugar metabolism, and porphyrin metabolism (represented by a metabolite from the heme group).

Lipidomic and metabolomic profiling provides a detailed view of sperm biochemistry. Lipidomics emphasize the structural and functional importance of membrane lipids, fatty acids, and sphingolipids in non-capacitated spermatozoa, while metabolomics confirm the active pathways related to lipid metabolism, steroid biosynthesis, and energy production. The convergence of these datasets demonstrates the robustness of our workflow and links structural lipids to their metabolic context, facilitating the interpretation of biological relevance.

To sum up, our optimized extraction and MS-based workflow substantially expands the current analytical coverage from minimal cellular input, providing a powerful platform for characterizing sperm biochemistry in both research and clinical contexts. Metabolomic analyses further confirmed active biochemical pathways associated with lipid metabolism, steroid biosynthesis, and energy production, corroborating the metabolic complexity and functional specialization of spermatozoa. Collectively, these findings demonstrate that untargeted lipidomics and metabolomics can capture molecular processes that are essential for sperm functionality. This offers new opportunities to identify biomarkers that can predict male fertility status. Future studies applying this method to larger and phenotypically stratified datasets may enable the identification of lipidomic and metabolomic signatures associated with male infertility, ultimately contributing to improved diagnostic accuracy and treatment selection.

## 5. Conclusions

This study presents an optimized HPLC-ESI-MS/MS workflow that enables comprehensive lipidomic and metabolomic profiling of human spermatozoa from a minimal number of cells. By integrating methodological improvements, including efficient sonication-based extraction and standardized dual normalization, this approach achieved the highest lipid and metabolite coverage reported to date in sperm cells. The resulting datasets reveal that the sperm lipidome is dominated by membrane-related lipids, such as cholesterol and glycerophospholipids, which are essential for membrane fluidity, integrity, and signaling. Given its efficiency and scalability, this workflow provides a powerful platform for translational research and future clinical implementation, particularly in the analysis of samples with limited sperm availability, helping to bridge the gap between molecular understanding and fertility diagnostics.

## Figures and Tables

**Table 1:** Summary of previously reported lipidomic studies performed in spermatozoa, including the technique, number of identified lipids, sperm counts, lipids identified per million sperm cells and references (* the total number of 479 lipids reported includes both techniques; the original publication does not specify the contribution of each individual method).

Technique	Identifications	Sperm Number (M)	Lipids per Million Cells	Reference
**HPLC-ESI-MS/MS**	**473**	**1.25**	**378.4**	**Our work**
(NP)-HPLC-MS RP- HPLC/MRM-MS	479 *	10	47.9	[27]
DI-ESI-MS	399	5.46	73.1	[11]
HPLC-ESI-MS/MS	209	20	10.5	[28]
FIA-MS/MS (targeted)	140	464	0.3	[12]

**Table 2:** Summary of previously reported untargeted metabolomic studies performed in spermatozoa, including the technique, number of identified lipids, sperm counts, metabolites identified per million sperm cells and references.

Technique	Identifications	Sperm Number (M)	Metabolites per Million Cells	Reference
**HPLC-ESI-MS/MS**	**2.435 total** **955 known**	**1.25 M**	**764**	**Our work**
HPLC-ESI-MS/MS	112	20 M	5.6	[28]
NMR	42	150 M	0.3	[6]
GC-MS	27	15 M	1.8	
GC-MS	33	102.8 M	0.3	[13]
FIA-MS/MS (targeted)	31	464 M	0.1	[12]

## Data Availability

The original contributions presented in this study are included in the article/Supplementary Material. Further inquiries can be directed to the corresponding author.

## Supplementary Materials

The following supporting information can be downloaded at: https://www.mdpi.com/article/10.3390/cells15070649/s1, Figure S1: Integrated workflow for lipidomic and metabolomic sample preparation, extraction and analysis of human spermatozoa; Figure S2: Confocal microscopy images of sperm sonication efficiency. (A) Intact spermatozoa before sonication. (B) No intact spermatozoa after sonication (effective disruption). (C) Intact spermatozoa remaining after sonication (ineffective disruption). Scale bar 50 µm; Figure S3: Effect of spermatozoa concentration on lipidomic coverage, variability and reproducibility. (A) Total number of lipids identified in spermatozoa and (B) violin plot distribution of lipid concentration at three different sperm concentrations at all sperm concentrations. (C) Venn diagrams of the overlap of lipid species identified across four replicates (L1–L4) for each spermatozoa concentration; Figure S4: Effect of spermatozoa concentration on metabolomic coverage, variability and reproducibility. (A) Total number of known metabolites identified in spermatozoa and (B) violin plot distribution of lipid concentration at three different sperm concentrations at all sperm concentrations. (C) Venn diagrams of the overlap of lipid species identified across four replicates (M1–M4) for each spermatozoa concentration; Table S1: Lipid composition of internal standard mix. Table S2: Descriptive and clinical parameters of the semen samples. Clinical data, seminal and kinematic parameters of the three biological semen samples used for lipidomic and metabolomics profile. N: Normozoospermia; T: Teratozoospermia; AT Asthenoteratozoospermia; ART, Assisted reproductive technologies; IUI: Intrauterine Artificial Insemination; Table S3: Sonication results by changing intensity; Table S4: Sonication results by changing the number of cycles; Table S5: Sonication results with 2.5 million cells by changing number of cycles and time, and protein concentration measurement; Table S6: Lipidomic analysis dataset. (Excel file). (A) ESI+ dataset. (B) ESI− dataset. (C) Unified dataset; Table S7: Metabolomic analysis dataset. (Excel file). (A) ESI+ dataset. (B) ESI− dataset. (C) Unified dataset.

## Abbreviations

The following abbreviations are used in this manuscript:

12-ALA	12-Aminolauric Acid
AcCa	Acylcarnitine
Ali-Ald	Aliphatic Aldehydes
Andr	Androgens
Aro-Ald	Aromatic Aldehydes
ART	Assisted Reproductive Technology
BCF	Beat Cross Frequency
C	Concentration
CASA	Computer-Assisted Sperm Analysis
Cer	Ceramide
Chol	Cholesterol
Cyc-Carbs	Cyclic Carbohydrates
DG	Diacylglycerol
DHA	Docosahexaenoic Acid
DI-MS	Direct Infusion Mass Spectrometry
EDE	Epoxydocosapentaenoic Acid Derivatives
ESI	Electrospray Ionization
ESI+	Positive Electrospray Ionization Mode
ESI−	Negative Electrospray Ionization Mode
Est	Estrogens
FA	Fatty Acids
FIA-MS	Flow Injection Analysis Mass Spectrometry
GC-MS	Gas Chromatography–Mass Spectrometry
Glyco-PG	Glycosylphosphatidylglycerol
GPI	Glycosylphosphatidylinositol
GSL	Glycosphingolipids
HETE	Hydroxyeicosatetraenoic Acid Derivatives
HILIC	Hydrophilic Interaction Liquid Chromatography
HPLC	High-Performance Liquid Chromatography
Hex1Cer	Monohexosylceramide
Hex2Cer	Dihexosylceramide
Hex3Cer	Trihexosylceramide
ICSI	Intracytoplasmic Sperm Injection
IPA	Isopropanol
IUI	Intrauterine Artificial Insemination
IVF	In Vitro Fertilization
LC-MS	Liquid Chromatography–Mass Spectrometry
LIN	Linearity Index
LPA	Lysophosphatidic Acid
LPC	Lysophosphatidylcholine
LPCe	Lysophosphatidylcholine (ether/plasmalogen)
LPE	Lysophosphatidylethanolamine
LPG	Lysophosphatidylglycerol
LPS	Lysophosphatidylserine
LX	Lipoxins
Lyso SM	Lysosphingomyelin
*m*/*z*	Mass-to-Charge Ratio
MeOH	Methanol
M	Morphology
MRM	Multiple Reaction Monitoring
MS	Mass Spectrometry
MS/MS	Tandem Mass Spectrometry
N-GEA	N-Gondoylethanolamine
N-LG	N-Lauroylglycine
N-UG	N-Undecanoylglycine
NMR	Nuclear Magnetic Resonance
NP	Normal Phase
OxLs	Oxylipins
PC	Phosphatidylcholine
PCe	Phosphatidylcholine (ether)
PE	Phosphatidylethanolamine
PE(e/p)	Phosphatidylethanolamine (ether/plasmalogen)
PG	Phosphatidylglycerol
PGs	Prostaglandins
PI	Phosphatidylinositol
ppm	Parts per million
PR	Progressive motility
Prog	Progestogens
PS	Phosphatidylserine
PUFAs	Polyunsaturated Fatty Acids
R^2^	Coefficient of Determination
RP	Reverse Phase
SFAs	Saturated Fatty Acids
SM	Sphingomyelin
SMPDB	Small Molecule Pathway Database
SPH	Sphingosine
ST	Sulfatide
Ster	Steroids
STR	Straightness Index
TG	Triacylglycerol
TMS	Total Motile Spermatozoa
TSC	Total Sperm Count
TX	Thromboxanes
UHPLC	Ultra-High-Performance Liquid Chromatography
V	Vitality
VAP	Average Path Velocity
VCL	Curvilinear Velocity
VSL	Straight-Line Velocity
WHO	World Health Organization
WOB	Wobble Index
