# Supplementary material for: Lipidomic and Metabolomic Profiling on Low-Count Human Spermatozoa: A Robust and Reproducible Method for Untargeted HPLC-ESI-MS/MS-Based Approach
Source: Cells. 2026 Apr 5;15(7):649. doi: 10.3390/cells15070649 (PMC13072360; doi:10.3390/cells15070649)
Supplement: Supplementary file 1 [file cells-15-00649-s001.zip › cells-4195410-supplementary/Supplelemtary Information/Supplementary Figures.pdf]

Supplementary Figures

Lipidomic and metabolomic profiling on low count human spermatozoa: A robust and reproducible method for untargeted HPLC-ESI-MS/MS-based approach

Irune Calzado [1,2], Manu Araolaza [1,2], Mikel Albizuri [1,2], Ainize Odriozola [1,2], Iraia Muñoa-Hoyos [1,2], Iratxe Ajuria-Morentin [3] and Nerea Subirán [1,2,*]

[1] Faculty of Medicine and Nursing, University of the Basque Country, 48940 Leioa, Bizkaia, Spain
[2] Bizkaia Health Research Institute, 48903 Barakaldo, Bizkaia, Spain
[3] Galdakao-Usansolo Hospital, 48960 Galdakao, Bizkaia, Spain
* Correspondence: nerea.subiran@ehu.eus; Tel.: +34-946015673

Supplemental Figure S1

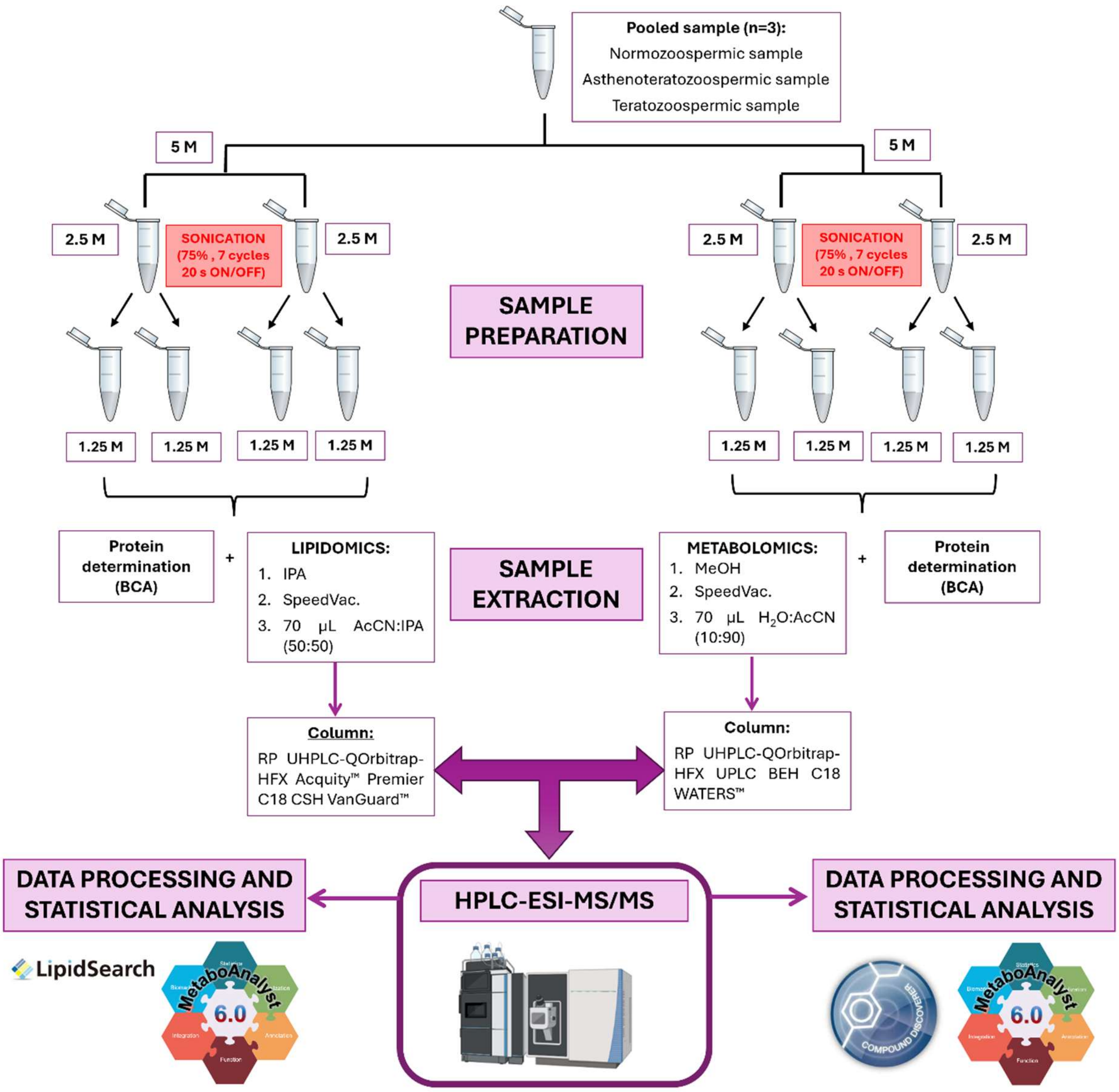

Supplemental Figure S1. Integrated workflow for lipidomic and metabolomic sample preparation, extraction and analysis of human spermatozoa.

Supplemental Figure S2

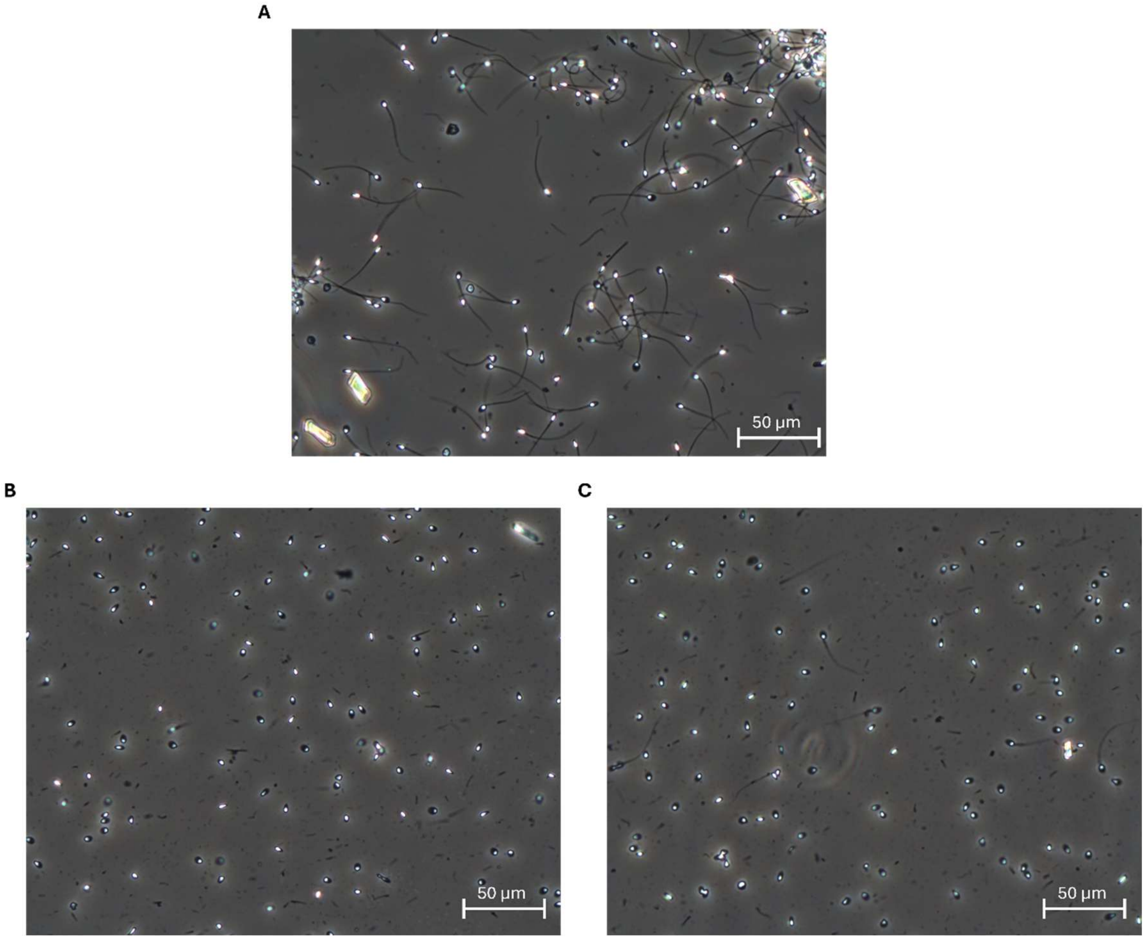

Supplemental Figure S2. Confocal microscopy images of sperm sonication efficiency. (A) Intact spermatozoa before sonication. (B) No intact spermatozoa after sonication (effective disruption). (C) Intact spermatozoa remaining after sonication (ineffective disruption). Scale bar 50 µm.

Supplemental Figure S3.

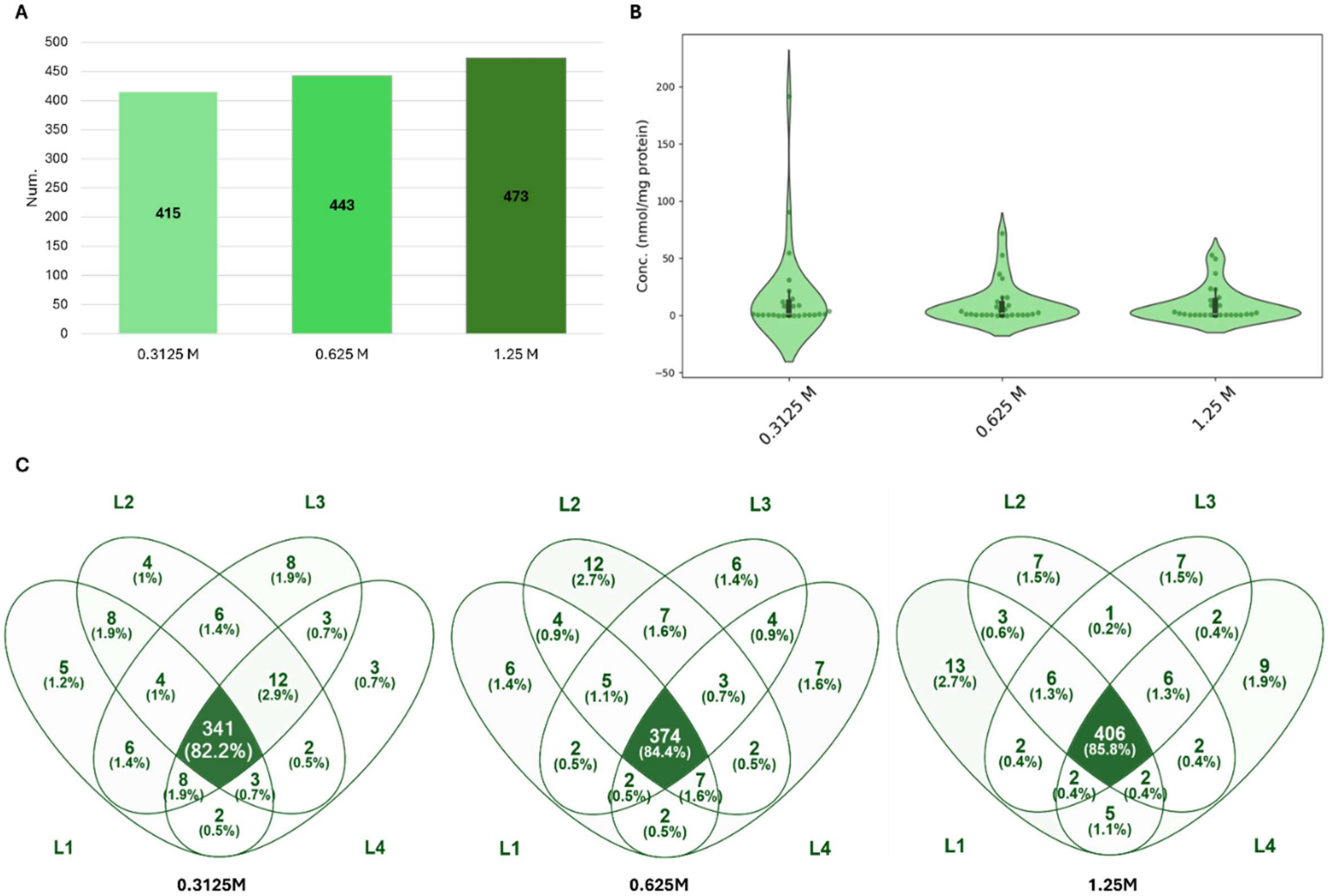

Supplemental Figure S3. Effect of spermatozoa concentration on lipidomic coverage, variability and reproducibility. (A) Total number of lipids identified in spermatozoa and (B) violin plot distribution of lipid concentration at three different sperm concentrations at all sperm concentrations. (C) Venn diagrams of the overlap of lipid species identified across four replicates (L1-L4) for each spermatozoa concentration.

Supplemental Figure S4.

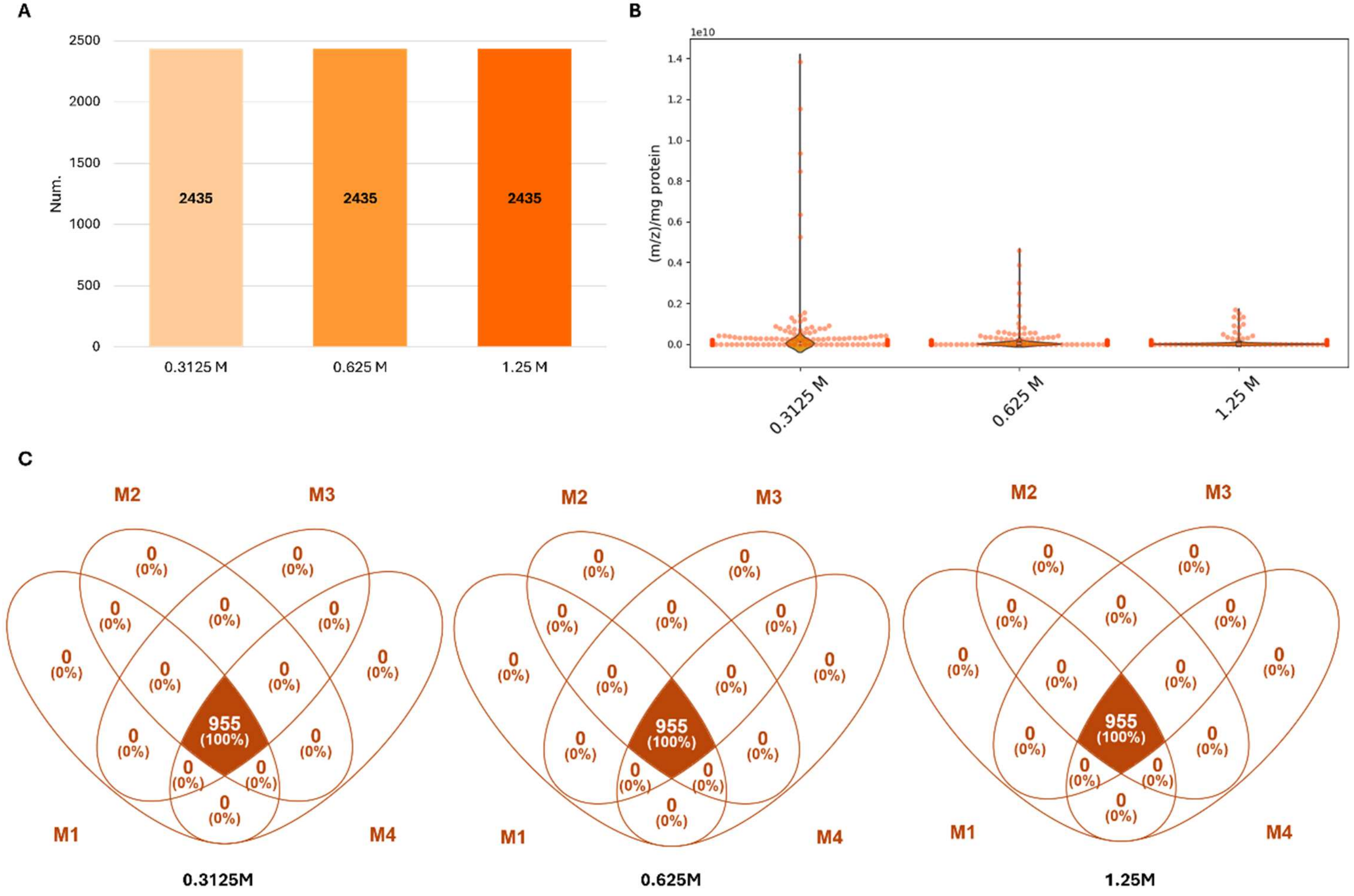

Supplemental Figure S4. Effect of spermatozoa concentration on metabolomic coverage, variability and reproducibility. (A) Total number of known metabolites identified in spermatozoa and (B) violin plot distribution of lipid concentration at three different sperm concentrations at all sperm concentrations. (C) Venn diagrams of the overlap of lipid species identified across four replicates (M1-M4) for each spermatozoa concentration.
