# Supplementary material for: Lipidomic and Metabolomic Profiling on Low-Count Human Spermatozoa: A Robust and Reproducible Method for Untargeted HPLC-ESI-MS/MS-Based Approach
Source: Cells. 2026 Apr 5;15(7):649. doi: 10.3390/cells15070649 (PMC13072360; doi:10.3390/cells15070649)
Supplement: Supplementary file 1 [file cells-15-00649-s001.zip › cells-4195410-supplementary/Supplelemtary Information/Supplementary Tables.pdf]

Supplementary Tables

Lipidomic and metabolomic profiling on low count human spermatozoa: A robust and reproducible method for untargeted HPLC-ESI-MS/MS-based approach

Irune Calzado [1,2], Manu Araolaza [1,2], Mikel Albizuri [1,2], Ainize Odriozola [1,2], Iraia Muñoa-Hoyos [1,2], Iratxe Ajuria-Morentin [3] and Nerea Subirán [1,2,*]

[1] Faculty of Medicine and Nursing, University of the Basque Country, 48940 Leioa, Bizkaia, Spain
[2] Bizkaia Health Research Institute, 48903 Barakaldo, Bizkaia, Spain
[3] Galdakao-Usansolo Hospital, 48960 Galdakao, Bizkaia, Spain
* Correspondence: nerea.subiran@ehu.eus; Tel.: +34-946015673

Supplemental Table S1. Lipid composition of internal standard mix.

Internal Standard Mix	Compound Name	Exact Mass	Chemical Formula	Conc. (µg/mL)	Conc. (µM)	PM	Conc. (µM)	Cant. (nmol)
Splash LipidoMix	15:0-18:1(d7) PC	752.6061	C41H73D7NO8P	150.6	200.0	753.11	199.97	2.00
Avanti 330707	15:0-18:1(d7) PE	710.5591	C38H67D7NO8P	5.3	7.5	711.03	7.45	0.07
(Avanti Polar Lipids)	15:0-18:1(d7) PS (Na Salt)	754.549	C39H67D7NO10P	3.9	5.0	777.02	5.02	0.05
	15:0-18:1(d7) PG (Na Salt)	741.5537	C39H68D7O10P	26.7	34.9	764.02	34.95	0.35
	15:0-18:1(d7) PI (NH4 Salt)	829.5698	C42H72D7O13P	8.5	10.0	847.13	10.03	0.10
	15:0-18:1(d7) PA (Na Salt)	667.5181	C36H61D7NaO8P	6.9	10.0	689.94	10.00	0.10
	18:1(d7) Lyso PC	528.3921	C26H45D7NO7P	23.8	45.0	528.72	45.01	0.45
	18:1(d7) Lyso PE	486.3451	C23H39D7NO7P	4.9	10.1	486.64	10.07	0.10
	18:1(d7) Chol Ester	657.6441	C45H71D7O2	329.1	500.0	658.16	500.03	5.00
	18:1(d7) MG	363.3366	C21H33D7O4	1.8	5.0	363.59	4.95	0.05
	15:0-18:1(d7) DG	587.5506	C36H61D7O5	8.8	15.0	587.98	14.97	0.15
	15:0-18:1(d7) -15:0 TG	811.7646	C51H89D7O6	52.8	65.0	812.37	65.00	0.65
	D18:1-18:1(d9) SM	737.6397	C41H72D9N2O6P	29.6	40.1	738.12	40.10	0.40
	Cholesterol (d7)	393.3988	C27H39D7O	98.4	249.9	393.71	249.93	2.50
Ceramide/Sphingoid Internal Standard Mixture I	Sphingosine (C17)	285.2668	C17H35NO2	7.1	25.0	285.4653	25.0	0.25
Avanti LM-6002	Sphinganine (C17)	287.2824	C17H37NO2	7.15	25.0	287.4812	25.0	0.25
	Sphingosine-1-PO4 (C17)	365.2331	C17H36NO5P	9.09	25.0	365.4452	25.0	0.25
	Sphinganine-1-PO4 (C17)	367.2488	C17H38NO5P	9.11	25.0	367.4611	25.0	0.25
	Ceramide (C12)	481.4495	C30H59NO3	12.42	25.0	481.7944	25.0	0.25
	Sphingomyelin (C12)	646.505	C35H71N2O6P	16.68	25.0	646.9218	25.0	0.25
	Glucosyl(β) C12 Ceramide	643.5023	C36H69NO8	16.34	25.0	643.935	25.0	0.25
	Lactosyl(β) C12 Ceramide	805.555	C42H79NO13	20.94	25.0	806.0756	25.0	0.25
Avanti 870857P (Avanti Polar Lipids)	24:0(d4) L-carnitine	515.48462	C31H57D4NO4	50	96.9	515.86	96.93	0.97

Supplemental Table S2. Descriptive and clinical parameters of the semen samples. Clinical data, seminal and kinematic parameters of the three biological semen samples used for lipidomic and metabolomics profile. N: Normozoospermia; T: Teratozoospermia; AT Asthenoteratozoospermia; ART, Assisted reproductive technologies; IUI: Intrauterine Artificial Insemination.

	N	T	AT	Mean	DV	Reference Value
**Clinical data**						
**Age (years)**	34	35	33	34	0.58	
**Smoking status**	Ex-smoker	Ex-smoker	Smoker 17 cig/day			
**ART-related diagnoses**	4 IUI cycles	Not ART-related diagnosis	2 IUI cycles			
**Seminal parameters**						
**Abstinence days**	5	4	5	4.67	0.33	
**Concentration (C_M/mL)**	195.84	200.17	25.78	140.60	57.49	≥39 M/sample
**Total sperm count (TSC_M)**	362.30	400.34	85.07	282.57	99.48	≥16 M/mL
**Progressive motility (PR_%)**	60.71	31.99	10.80	34.50	14.48	≥30 %
**Total motile spermatozoa (TMS_%)**	70.65	46.29	18.08	45.01	15.21	≥42 %
**Morphology (M_%)**	31.40	0.91	2.54	11.62	9.91	≥4 %
**Vitality (V_%)**	86	69	57.5	70.83	8.29	≥54 %
**Kinematic parameters**						
**Curvilinear Velocity (VCL_ μm/s)**	77.52	40.34	43.71	53.86	11.89	
**Straight-Line Velocity (VSL_μm/s)**	61.01	26.79	25.53	37.78	11.64	
**Average Path Velocity (VAP_μm/s)**	65.74	30.59	30.02	42.12	11.83	
**Linearity Index (LIN_%)**	70.22	58.74	45.15	58.04	7.25	
**Straightness Index (STR_%)**	84.23	77.55	67.17	76.32	4.97	
**Wobble Index (WOB_%)**	79.93	71.84	59.11	70.29	6.07	
**Amplitude of Lateral Head (ALH_μm)**	2.13	1.65	1.88	1.89	0.14	
**Beat Cross Frequency (BCF_Hz)**	9.04	7.71	5.44	7.40	1.05	
**Hyperactivated spermatozoa (%)**	0	0	0	0.00	0.00	

Supplemental Table S3. Sonication results by changing intensity.

Sonication conditions		*3 cycles of 10s ON/10s OFF*	
Aliquot	**Intensity (%)**	**Sperm cells (M)**	**Broken spermatozoa?**
1	25	1	☒
2	25	1	☒
3	30	1	☒
4	40	1	☒
5	60	1	☒
6	75	1	☑

Supplemental Table S4. Sonication results by changing the number of cycles.

Sonication conditions			*Intensity 75 %, different cycles of 10s ON/10s OFF*	
Aliquot	**Sperm cells (M)**	**Cycles**	**Total time ON (s)**	**Broken spermatozoa?**
1	1.43	3	30	☑
2	1.43	3	30	☑
3	1.43	5	50	☑
4	1.43	5	50	☑
5	1.43	7	70	☑
6	1.43	7	70	☑

Supplemental Table S5. Sonication results with 2.5 million cells by changing number of cycles and time, and protein concentration measurement.

Sonication conditions				*Intensity 75 %, 2.5 M sperm cells*	
Aliquot	*Cycles*	*Time ON/OFF (s)*	*Total time ON*	*Broken spermatozoa?*	*Protein concentration (µg/mL)*
**1**	*7*	10	1 min 10 s	☒	170
**2**	*7*	10	1 min 10 s	☒	140
**3**	*4*	20	1 min 20 s	☑	150
**4**	*4*	20	1 min 20 s	☒	130
**5**	*5*	20	1 min 40 s	☑	130
**6**	*5*	20	1 min 40 s	☑	140
**7**	*5*	20	1 min 40 s	☒	170
**8**	*5*	20	1 min 40 s	☒	100
**9**	*7*	20	2 min 20 s	☑	150
**10**	*7*	20	2 min 20 s	☑	170

Supplemental Table S6. Lipidomic analysis dataset. (Excel file)

(A) ESI+ dataset.
(B) ESI- dataset.
(C) Unified dataset.

Supplemental Table S7. Metabolomic analysis dataset. (Excel file)

(A) ESI+ dataset.
(B) ESI- dataset.
(C) Unified dataset.
